# Diet Modifies Pioglitazone's Influence on Hepatic PPAR*γ*-Regulated Mitochondrial Gene Expression

**DOI:** 10.1155/2020/3817573

**Published:** 2020-09-10

**Authors:** Sakil Kulkarni, Jiansheng Huang, Eric Tycksen, Paul F. Cliften, David A. Rudnick

**Affiliations:** ^1^Department of Pediatrics, Washington University School of Medicine, St. Louis, MO 63110, USA; ^2^Department of Genetics, Washington University School of Medicine, St. Louis, MO 63110, USA; ^3^Department of Developmental Biology, Washington University School of Medicine, St. Louis, MO 63110, USA

## Abstract

Pioglitazone (Pio) is a thiazolidinedione (TZD) insulin-sensitizing drug whose effects result predominantly from its modulation of the transcriptional activity of peroxisome proliferator-activated-receptor-gamma (PPAR*γ*). Pio is used to treat human insulin-resistant diabetes and also frequently considered for treatment of nonalcoholic steatohepatitis (NASH). In both settings, Pio's beneficial effects are believed to result primarily from its actions on adipose PPAR*γ* activity, which improves insulin sensitivity and reduces the delivery of fatty acids to the liver. Nevertheless, a recent clinical trial showed variable efficacy of Pio in human NASH. Hepatocytes also express PPAR*γ*, and such expression increases with insulin resistance and in nonalcoholic fatty liver disease (NAFLD). Furthermore, mice that overexpress hepatocellular PPAR*γ* and Pio-treated mice with extrahepatic PPAR*γ* gene disruption develop features of NAFLD. Thus, Pio's direct impact on hepatocellular gene expression might also be a determinant of this drug's ultimate influence on insulin resistance and NAFLD. Previous studies have characterized Pio's PPAR*γ*-dependent effects on hepatic expression of specific adipogenic, lipogenic, and other metabolic genes. However, such transcriptional regulation has not been comprehensively assessed. The studies reported here address that consideration by genome-wide comparisons of Pio's hepatic transcriptional effects in wildtype (WT) and liver-specific PPAR*γ*-knockout (KO) mice given either control or high-fat (HFD) diets. The results identify a large set of hepatic genes for which Pio's liver PPAR*γ*-dependent transcriptional effects are concordant with its effects on RXR-DNA binding in WT mice. These data also show that HFD modifies Pio's influence on a subset of such transcriptional regulation. Finally, our findings reveal a broader influence of Pio on PPAR*γ*-dependent hepatic expression of nuclear genes encoding mitochondrial proteins than previously recognized. Taken together, these studies provide new insights about the tissue-specific mechanisms by which Pio affects hepatic gene expression and the broad scope of this drug's influence on such regulation.

## 1. Introduction

Pioglitazone (Pio) is a thiazolidinedione (TZD) agonist of the nuclear hormone receptor (NHR) peroxisome proliferator-activated receptor gamma (PPAR*γ*). Pio-bound PPAR*γ* forms a heterodimer with retinoid X receptor (RXR [1]), and the PPAR*γ*-RXR complex interacts with specific peroxisome proliferator response elements (PPREs) in DNA to regulate metabolic and other gene expressions in adipose and other tissues [2]. Pio has beneficial effects on insulin sensitivity and nonalcoholic fatty liver disease (NAFLD) in experimental animal models [3–7], and this drug is approved for treatment of insulin-resistant diabetes in humans [8]. Pio's efficacy in human nonalcoholic steatohepatitis (NASH), which is strongly associated with insulin resistance, was recently investigated in the “**Pi**oglitazone versus **V**itamin **E** versus Placebo for the Treatment of Non-Diabetic Patients with **N**onalcoholic **S**teatohepatitis” (i.e. **PIVENS**) clinical trial [9]. The results showed favorable Pio-induced changes in NASH activity in some study subjects. However, most Pio-treated PIVENS subjects did not exhibit benefit from that treatment. These observations implicate still-unknown genetic and/or environmental factors as modifiers of Pio's physiological effects in mice and humans. Other research has revealed tissue-specific effects of PPAR*γ*, the canonical target of Pio, on NAFLD in experimental models. For example, hepatocellular PPAR*γ* overexpression induces hepatic steatosis [10], while liver-specific PPAR*γ* disruption prevents hepatic fat accumulation in mouse NAFLD models [11–13]. Conversely, muscle- [14] or adipocyte-specific PPAR*γ* deletion promotes hepatic steatosis [15], which is consistent with the idea that PPAR*γ* activity in adipose, and perhaps muscle, mediates much of Pio's efficacy towards NAFLD [16]. Other studies show that hepatic PPAR*γ* expression, though lower than that of hepatic PPAR*α* expression, is nevertheless significantly induced in experimental and human NAFLD [17–19]. Taken together, these considerations raise the interesting possibility that Pio's variable influence on human NAFLD in the PIVENS trial [9] could have resulted at least in part from the effects of genetic or environmental modifiers on hepatic PPAR*γ* expression and/or transcriptional activity.

PPAR*γ*-independent effects of Pio have also been implicated as candidate mediators of Pio's beneficial effects on hepatic steatosis in experimental models. For example, direct effects of this drug on the mitochondrial pyruvate carrier protein (MPC) were recently identified as a possible contributor to Pio's beneficial effects in NAFLD [20, 21]. Indeed, that consideration has stimulated new interest in developing PPAR*γ*-sparing TZDs as novel pharmacological approaches to human NAFLD that avoid TZD-associated side effects. Other studies have identified beneficial effects of both Pio and PPAR*γ* on mitochondrial function in experimental models of NAFLD [22] and other diseases [23]. Although Pio's PPAR*γ*-dependent effects on hepatic expression of specific adipogenic and other genes have been investigated [10, 12, 13, 24], comprehensive assessments of such regulation in general and of hepatic mitochondrial gene expression in particular have not been reported. Moreover, whether genetic or environmental modifiers influence such control remains unknown. The studies reported here address these considerations by testing the hypothesis that diet alters Pio-induced, liver-PPAR*γ*-dependent regulation of hepatic gene expression and comprehensively characterizing such regulation in mice. Our results provide new insights into the tissue-specific mechanisms by which Pio and hepatocellular PPAR*γ* interact to influence hepatic gene expression and have implications for the variable efficacy Pio had in the PIVENS trial.

## 2. Experimental Methods

### 2.1. Mouse Husbandry

Liver-specific PPAR*γ* knockout (KO) mice were generated by breeding PPAR*γ*-loxP mice (The Jackson Laboratory, Bar Harbor, ME) to transgenic Alb-Cre (B6.Cg-Tg (Alb-Cre) 21Mgn/J, The Jackson Laboratory) mice as previously described [25]. Two-month-old male KO or control (i.e., wildtype (WT) C57BL6/J; The Jackson Laboratory) mice were given ad lib access to sterile, irradiated low-fat (S4031, BioServ, Flemington, NJ) or high-fat (S3282, BioServ; 60% kcal from fat) diets, with or without 0.01% (*w*/*w*) pioglitazone hydrochloride (Pio, Sigma-Aldrich, St. Louis, MO) for 3 months (n = 4 − 5 mice/experimental group). Body weight was measured weekly and body composition analyzed at the experimental endpoint by ECHO MRI spectroscopy [26]; after which, mice were euthanized for tissue harvest and analysis. Blood glucose, serum insulin and free fatty acid levels, and liver histology and triglyceride content were determined as previously described [25–27]. All experiments were approved by the Washington University Institutional Animal Care and Use Committee (IACUC), and all animals received humane care in accordance with institutional guidelines and criteria in the “Guide for the Care and Use of Laboratory Animals” (8^th^ edition, 2011, https://grants.nih.gov/grants/olaw/guide-for-the-care-and-use-of-laboratory-animals.pdf).

### 2.2. Hepatic Transcriptomic Analyses (RNA-Seq and RT-qPCR)

Total liver RNA was purified using the Trizol method (Invitrogen, Carlsbad, CA). Libraries were prepared by poly-A selection of 10 *μ*g of liver RNA using Invitrogen mRNA Direct™ kits. mRNA was fragmented for cDNA synthesis, which was done using Invitrogen Superscript III and random hexamers. The cDNAs were end repaired, A-tailed, and ligated to standard Illumina adapters. Libraries were amplified with primers to incorporate a unique index into each sample. RNA-seq reads were aligned to the Ensembl release 76 GRCm38 assembly with STAR version 2.0.4b. Gene counts were derived from the number of uniquely aligned unambiguous reads by Subread:featureCount version 1.4.5.

All gene-level counts were then imported into R version 3.4.1, and TMM normalization size factors were calculated to adjust samples for differences in library size with the Bioconductor package EdgeR version 3.20.2. Genes expressed less than 1 count per million in less than 5 samples, and ribosomal genes were excluded from further analysis. The TMM size factors and the matrix of counts were then imported into the R/Bioconductor package Limma version 3.34.4 [28, 29], and weighted likelihoods based on the observed mean-variance relationship of every gene were then calculated for all samples with the voomWithQualityWeights function. Performance of the samples was then assessed with Spearman correlation matrix multidimensional scaling plots and principal component analysis. Gene performance was assessed with plots of residual standard deviation of every gene to their average log-count with a robustly fitted trend line of the residuals. Generalized linear models with robust dispersion estimates were then created to test for gene/transcript level differential expression. Differentially expressed genes and transcripts were then filtered for FDR adjusted *p* values of *q* ≤ 0.05 [30, 31]. To enhance the biological interpretation of the large set of transcripts, the filtered gene lists were interrogated for overrepresentation of Hallmark gene sets using the hypergeometric tests available through the Broad Institute's Molecular Signature Database (http://software.broadinstitute.org/gsea/msigdb/annotate.jsp [32, 33],). Perturbations in expression across these gene sets versus the background gene signals were assessed with the R package Gage version 2.28.0.

Hepatic expression patterns of some of the genes identified as differentially expressed by RNA-Seq were confirmed using real-time semiquantitative RT-PCR (RT-qPCR) analyses as described previously [27] together with the oligonucleotide primers listed in Supplementary Table S1.

### 2.3. RXR Chromatin Immunoprecipitation (ChIP) with High Throughput DNA Sequencing (Seq)

RXR ChIP-Seq was conducted using an approach analogous to the one previously employed for acetyl-histone ChIP-Seq [27, 34]. Briefly, frozen liver was minced, crosslinked in 1% formaldehyde, homogenized, and suspended in nuclear lysis buffer in the presence of protease inhibitors, then centrifuged to recover chromatin, which was sheared in a Bioruptor Sonicator (Diagenode, Denville, NJ) to generate uniform 100-500 base pair (bp) fragments. Equal quantities of chromatin (based on protein content) were immunoprecipitated using a ChIP-grade anti-RXR*α*/*β*/*γ* antibody (sc-774; Santa Cruz Biotechnology, Inc., Beverly, MA) previously validated and used in RXR ChIP-Seq studies of liver tissue [35, 36]. The studies reported here adhered to the guidelines and practices recommended for analyses and quality control of ChIP-Seq data by the Encyclopedia of DNA elements (i.e., ENCODE and modENCODE) consortia guidelines [37]. These include recommendations to validate the specificity of the ChIP target transcription factor antibody by immunoblot (Supplementary Figure S1) and to assess each replicate of immunoprecipitated chromatin for quality metrics including NSC, RSC, Qtag score, and Irreproducible Discovery Rate (IDR) data. As described in Supplementary Materials, assessment of those metrics here met ENCODE guidelines' criteria (as described in detail in the Supporting Information). Of note, the replicate livers studied here came from separate animals as opposed to the independent cell cultures, embryo pools, or tissue sampling that account for most substrates of the ENCODE experiments. Based on that, we anticipated the possibility of increased variability when considering experimental design, which prompted us to use liver samples from each of 5 mouse replicates per group, retain all replicates for these analyses, and apply the additional stringencies to data analysis described below.

Immunoprecipitated DNAs and corresponding input samples were submitted to the WU GTAC for blunt ending, adaptor ligation, size selection, and amplification according to established protocols and as previously described [27, 34]. Libraries were sequenced using the Illumina HiSeq-3000 as single 50 bp reads. Raw data were demultiplexed and aligned to the most recent mouse reference genome assembly (i.e., mm10) using Novoalign (Novocraft; Selangor, Malaysia). Sequence peaks were identified by comparing data from the anti-RXR antibody immunoprecipitated samples to corresponding inputs for each replicate using MACS2 [38]. Peaks were associated to genes using Peak Annotation and VISualization (PAVIS) software [39]. Significant differences in gene-associated peak sequence abundances between experimental groups were determined using DiffBind, an open source Bioconductor package that utilizes edgeR software for statistical analysis of replicated sequence count data [30, 31]. These analyses used a Benjamini and Hochberg false discovery rate (FDR) threshold of *q* < 0.05 [40, 41]. We also applied the following additional stringencies: (i) ≥2-fold change in RXR liver-DNA binding between experimental groups as defined by DiffBind and (ii) identification of the gene-associated RXR-bound peak by MACS in at least 3 (out of the 4-5) replicates in each experimental group with increased binding. Gene set overrepresentation analysis was conducted on genes differentially bound by RXR using the approach described for RNA-Seq data analyses.

Efforts to conduct PPAR*γ* ChIP-Seq analyses on these liver samples were also attempted here, but those efforts were unsuccessful based on inability to identify a PPAR*γ* antibody meeting the ENCODE guidelines when tested on mouse liver (as summarized above and described in detail in the Supporting Information; S. Kulkarni, J. Huang, and D.A. Rudnick, unpublished observations). Therefore, in order to assess the liver PPAR*γ*-dependence of RXR liver DNA binding in the samples studied here, RXR-ChIP-Seq studies were conducted on livers from WT and liver-specific PPAR*γ* KO mice and the result compared.

### 2.4. Venn Diagram Analyses and Binding and Expression Target Analysis (BETA)

Interactivenn [42]) was used to determine and illustrate overlaps between genes identified as differentially expressed or differentially RXR-bound between groups. RXR ChIP-Seq and RNA-Seq datasets were also compared using “Binding and Expression Target Analysis” (BETA) software [43]. BETA is a publicly available software package that integrates ChIP- and RNA-Seq data to infer the function of a cis-acting regulator (in this case RXR-DNA binding as defined by ChIP-Seq data) on gene-specific patterns of expression (as defined by RNA-Seq data). Here, RXR-ChIP-Seq-defined peaks identified as differentially RXR-bound between groups (*q* < 0.05) were entered into the BETA algorithm (as a bed file using the format: chromosome number, chromosome start locus, and chromosome end locus). Differential gene expression data, extracted from the RNA-Seq data analysis, was provided to the software (as a tab-delimited text file using the format: gene ID, expression change, and FDR). The BETA algorithm assigned a rank and rank-product to each gene, with higher ranks and lower rank-products corresponding to genes whose change in expression is more likely to be regulated by the transcription factor-DNA binding event. Inferences about the effects of Pio, hepatic PPAR*γ* expression, and HFD exposure on RXR-dependent liver gene expression were made based on comparisons between experimental groups.

### 2.5. Statistical Analysis

ChIP- and RNA-Seq datasets were analyzed as described above. All other data were analyzed using SigmaPlot 13.0 (Systat Software Inc., San Jose, CA). Numerical data comparisons between groups were made using unpaired two-tailed Student's *t*-test for pair-wise comparisons and ANOVA for multiple groups. Secondary post hoc comparisons were conducted using Holm-Sidak for normally distributed data or Tukey for data that was not normally distributed. Chi-squared analysis was used to compare rates and proportions between groups. Significance (alpha) was set at 0.05. Data are reported as mean ± standard error.

## 3. Results

### 3.1. Pio Has Distinct Effects on Mouse Metabolism in WT versus Liver-Specific PPAR*γ* KO Mice Given Control versus HFD

**Figure 1:**
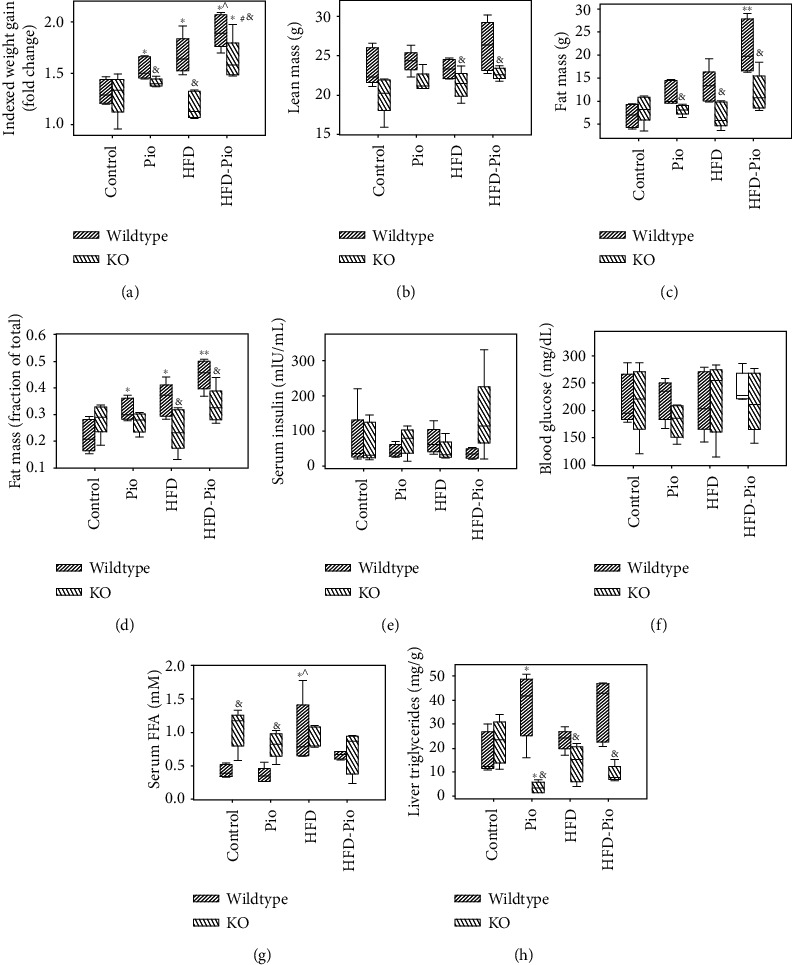
Effects of Pio on WT and PPAR*γ* KO mice. (a) Weight (indexed to initial weight), (b) lean mass (g), (c) fat mass (g), (d) fat mass (expressed as fraction of total mass), (e) serum insulin (mIU/mL, nonfasting a.m.), (f) blood glucose (mg/dL, nonfasting a.m.), (g) serum free fatty acids (FFA) (mM, nonfasting a.m.), and (h) liver triglycerides (mg/g liver) in wildtype (WT) and liver-specific PPAR*γ* knockout (KO) mice given a control or high-fat diet (HFD) unsupplemented or supplemented with Pioglitazone (Pio). ^∗^*p* < 0.05 vs. corresponding control diet-treated group; ^∧^*p* < 0.05 vs. corresponding Pio-treated group; ^∗∗^*p* < 0.05 vs. all other WT groups; ^#^*p* < 0.05 vs. corresponding HFD-treated group; ^&^*p* < 0.05 vs. corresponding WT group.

**Figure 2:**
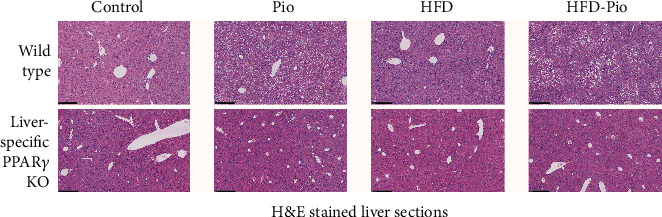
Effects of Pio on WT and KO mouse liver histology. H&E -stained liver sections from control diet (Control)-, Pio-, high-fat diet (HFD)-, or HFD and PIO (HFD-Pio)-treated mice. 500 micron bar shown in lower left corner.

Unsupplemented- or Pio-supplemented-control (i.e., “control” or “Pio”) or high-fat (i.e., “HFD” or “HFD-Pio”) diets were given to male WT and liver-specific PPAR*γ* KO mice from age 2 to 5 months. Initial body weights were comparable between treatment groups (i.e., ±Pio, ±HFD) but significantly greater in 2-month-old WT versus age-matched KO mice (Supplementary Figure S2). By the experimental endpoint, 5-month-old WT mice exposed to Pio, HFD, or HFD-Pio showed significantly greater weight gain and body fat mass fractions compared to controls (Figures 1(a)–1(d)). In contrast, KO mice displayed only limited changes in weight and no change in percent body fat mass in response to either intervention. Consistent with those findings, lean mass, fat mass, and percent fat mass were also significantly higher in HFD and HFD-Pio-treated WT mice compared to the corresponding KOs. Nonfasting a.m. serum insulin and blood glucose levels were comparable across all experimental groups (Figures 1(e)–1(f)). HFD-exposed WT mice displayed increased serum-free fatty acids (FFA's) compared to control and Pio-treated WT mice, while control and Pio-treated KO mice exhibited increased serum FFA's compared to the corresponding WT animals (Figure 1(g)). Finally, the effects of both Pio and diet on liver triglyceride content and hepatic steatosis varied depending on hepatocellular PPAR*γ* expression: Pio increased liver fat content in WT mice on control diet but decreased these measures in the corresponding KO mice, and HFD-exposed KO mice displayed reduced liver triglycerides and less hepatic steatosis than the corresponding WT mice (Figures 1(h) and 2). Taken together, these data reveal distinct hepatic and systemic metabolic responses to Pio in the absence versus the presence of HFD and define the hepatocellular PPAR*γ* dependence of those effects.

### 3.2. Diet Modifies Some of the Effects of Pio on Hepatic Gene Expression

**Figure 3:**
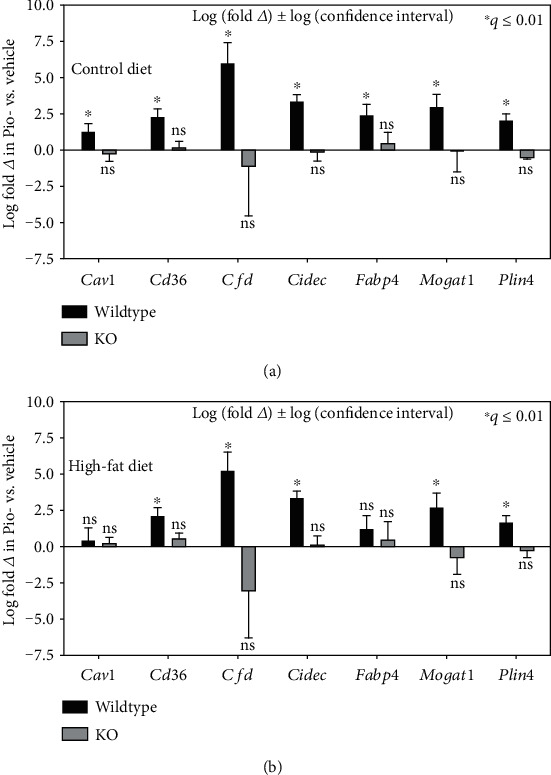
Effects of Pio on hepatic expression of exemplar genes. Summary of RNA-Seq data of exemplar genes whose expression is known to be regulated by PPAR*γ*. ^∗^*q* ≤ 0.01; ns: no significant difference.

Next, Pio's influence on genome-wide patterns of hepatic gene expression in WT mice on control or HFD was determined, with those results compared to corresponding analyses of the liver-specific PPAR*γ* KO mice. First, to establish the validity of this approach, these transcriptomic data were first inspected for patterns of Pio-induced, PPAR*γ*-dependent changes in hepatic expression of canonical adipogenic and lipogenic genes whose PPAR*γ*-regulated hepatic expression has previously been reported [10, 12, 13, 24]. Consistent with such published data, the transcriptomic data reported here show Pio-induced hepatic expression of Caveolin 1 (*Cav1*), *Cd36*, Complement factor D (*Cfd*, also known as Adipsin), *Cidec* (also known as *Fsp27*), *Fabp4* (also known as *Ap2*), *Mogat1*, and *Plin4* (also known as *S3-12*) in WT but not KO mice on control diet (Figure 3(a)). Further analyses also showed that Pio induces *Cd36*, *Cfd*, *Cidec*, *Mogat1*, and *Plin4* but not *Cav1* or *Fabp4* in WT mice on the HFD. As for mice on the control diet, Pio also had no significant effects on expression of these genes in HFD-treated KO mice (Figure 3(b)). Finally, to further validate our transcriptomic data, the RNA-Seq based results depicted in Figure 3 were compared to RT-qPCR based analyses of differential expression between groups in these exemplar genes, with the results of the latter studies (Supplementary Figure S3) highly concordant with those of the former (Figure 3). These data are consistent with previously published analyses of PPAR*γ*-dependent hepatic transcriptional effects, and they implicate diet as a specific modifier of those effects.

**Figure 4:**
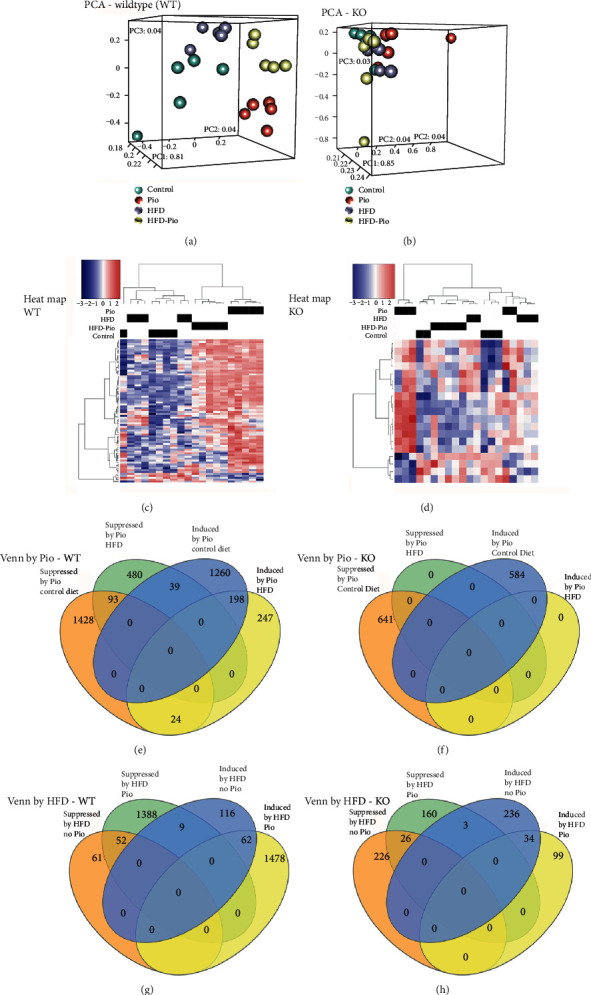
Effects of Pio on the hepatic transcriptome. (a, b) Principle component (PCA) and (c, d) heat map analyses of differentially expressed genes from RNA-Seq analyses of livers from (a, c) WT or (b, d) KO mice treated with control diet, Pio, HFD, or HFD and Pio. Heat map analyses are based on Pio-induced differences in gene expression. (e–h) Venn diagrams (generated using InteractiVenn [42]) depict overlap between differentially expressed genes by treatment group in WT (e, g) or KO (f, h) mice.

Next, these transcriptomic data were subjected to principle component analysis (PCA). The results showed excellent segregation of replicates of WT mice by exposure to drug (±Pio) and diet (±HFD) (Figure 4(a)). In contrast, the corresponding KO replicates showed poor separation (Figure 4(b)). Similarly, heat map analyses of Pio-induced changes in hepatic gene expression also showed more faithful segregation of the WT versus the KO experimental replicates (Figures 4(c) and 4(d)). Consistent with the exemplar gene expression data in Figure 3, Venn diagram analyses of patterns of overlap in these data identified subsets of genes whose Pio-induced hepatic expression is concordantly regulated in WT mice on control or HFD, and others whose Pio-induced regulation is altered by HFD (Figure 4(e)). This analysis also revealed a lack of concordance between the hepatic transcriptional effects of Pio in KO mice on control versus HFD and, furthermore, that Pio has no significant effects on hepatic gene expression in KO mice on HFD (Figure 4(f)). The results of the converse analyses, comparing the influence of diet on mice with or without Pio exposure, provide further support for the modifying influence of diet on Pio-induced changes in hepatic gene expression (Figures 4(g) and 4(h)). Taken together, these data reveal interactions between diet and hepatocellular PPAR*γ* expression that influence Pio-induced changes in hepatic gene expression.

To further explore dietary influences on Pio-regulated changes in hepatic gene expression, those genes identified as differentially expressed in the studies summarized in Figure 4 were subject to gene set overrepresentation analysis using the Hallmark gene set database, on the Broad Institute platform (http://software.broadinstitute.org/gsea/msigdb/annotate.jsp [32, 33],), and the most stringent FDR threshold cutoff on that platform (i.e., *q* < 1*e*^−6^). This evaluation identified significant enrichment of genes induced by Pio in livers from WT mice on the control diet for those associated with metabolic and other functional categories (Table 1(a)). The most highly enriched of those groupings, fatty acid metabolism, adipogenesis, and bile acid metabolism, were also identified as enriched for genes induced by Pio in WT mice on the HFD (Table 1(b)). However, this analysis also revealed distinct hepatic transcriptional effects of Pio in WT mice on control versus HFD, including specific enrichment of genes associated with cholesterol homeostasis in livers from mice on the control but not the HFD diet, and of glycolysis in mice on HFD but not control diet (Table 1(a) and (b)). These transcriptomic data were also subjected to GAGE analysis for gene set level changes in expression, and the results of that independent assessment of these data are highly concordant with those of the gene set overrepresentation study (Supplementary Table S3). For example, the GAGE examination also identified enrichment of Pio-induced genes associated with fatty acid metabolism, adipogenesis, and bile acid metabolism in mice on either diet, but specific enrichment for cholesterol homeostasis genes in mice on the control diet and for glycolysis genes in mice on the HFD. Genes whose hepatic expression is suppressed by Pio in WT mice were similarly assessed and also showed marked differences between control and HFD-treated mice. For example, those suppressed by Pio in WT mice on control diets are enriched in many different categories, including UV response up and heme metabolism, while those suppressed by Pio in mice on HFD were not significantly enriched for any gene categories when analyzed in this way (Table 1C and D). As for Pio-induced genes, the results of GAGE-based investigation of these data are also quite concordant with gene set overrepresentation analyses (Supplementary Table S3). Similar hepatic transcriptomic investigations of liver-specific PPAR*γ* KO mice, using gene set over-representation- and GAGE-based analyses, identified distinct dietary effects on patterns of Pio-induced and Pio-suppressed genes in these compared to WT mice (Table 2 vs. Table 1 and Supplementary Table S3). In this case, gene set overrepresentation analyses showed that Pio's suppressive effects on hepatic gene expression in KO mice on control diet and its inductive and suppressive effects in KO mice on HFD are virtually undetectable (Table 2B–D). Converse analyses, of the influence of diet in the absence or presence of Pio, showed a greater range of dietary influence in Pio-treated compared to untreated WT mice and markedly fewer effects of diet on the KO mice (Figures 4(g) and 4(h) and Supplementary Tables S4 and S5). Taken together, these data provide evidence for the modifying influence of diet on PPAR*γ*-dependent, Pio-induced changes in hepatic gene expression.

### 3.3. PPAR*γ*-Dependent Effects of Pio and HFD on Liver RXR-DNA Binding Are Highly Concordant with Corresponding Effects on Hepatic Gene Expression

**Figure 5:**
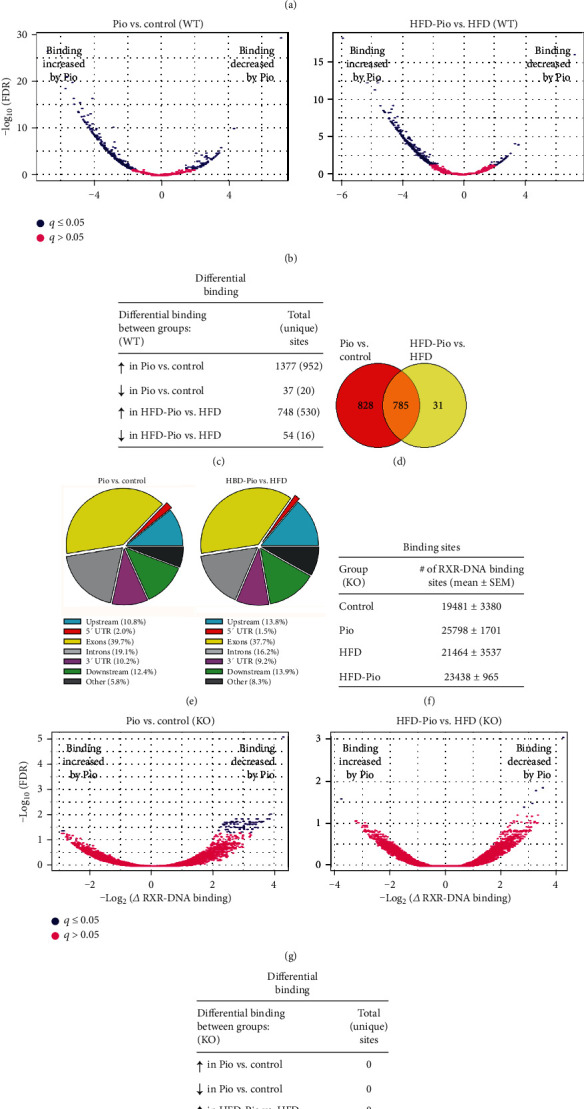
Effects of Pio on the hepatic RXR cistrome. (a) Total RXR-DNA binding sites (by group), (b) Volcano plots comparing differential RXR-DNA binding in replicate livers from Pio- versus Control- or HFD-Pio- versus HFD-treated groups of mice, and (c) Total (and unique) DNA sites with significantly different RXR-DNA binding (between groups), each defined as described in Experimental methods. (d) Venn diagram of overlap between sites of significant Pio-induced RXR-liver DNA binding in mice on control versus HFD. (e) Pie chart illustration of the locations of sites for genes summarized in (d) with respect to gene-specific transcription start sites in control versus HFD mice. (f–h) Results of corresponding RXR-liver DNA binding ChIP-Seq analyses of liver-specific PPAR*γ* KO mice.

To further characterize Pio's direct effects on hepatic transcriptional regulation, the consequences of Pio and HFD on gene-specific RXR-binding to liver DNA in WT and KO mice were characterized and compared by RXR-chromatin-immunoprecipitation combined with next-generation DNA sequencing (ChIP-Seq). The results identified specific RXR-DNA binding sites in both untreated and Pio-treated WT mice on either a control or HFD. They also revealed Pio-induced differences in such binding at some of those sites. In most cases, Pio increased RXR-DNA binding, but sites whose RXR-DNA binding is suppressed by Pio were also identified (Figures 5(a)–5(c)). This analysis also showed that Pio has broader effects on gene-specific RXR-liver DNA binding in WT mice on the control versus the HFD, with most binding sites identified in HFD-treated mice also identified in mice on the control diet but many binding sites detected in mice on the control diet not detected in those on the HFD (Figure 5(d)). Thus, these data provide further evidence for the modifying influence of diet on Pio-induced hepatic transcriptional regulation, and they implicate diet-induced differences in Pio's direct effects on liver RXR-DNA binding as a contributing mechanism. More than half of the Pio-induced RXR-liver DNA binding sites detected in WT mice on either diet occurred within exons or introns of specific genes, with binding to 5′ or 3′ untranslated regions (UTRs), upstream, downstream, or other sites accounting for the rest of such binding (Figure 5(e)). While parallel analyses of the KO mice also identified RXR-liver DNA binding sites (Figure 5(f)), in this case, very few sites with significant (i.e., *q* < 0.05) Pio-induced changes in RXR binding were identified (Figure 5(g)). Furthermore, most of those sites showed Pio-inhibited binding (Figure 5(g)) and none withstood the additional stringencies of our analytical approach (Figure 5(h) and Experimental Methods). Further analyses of HFD effects on such regulation identified a small number of differentially bound genes in WT mice in the absence of Pio and none in WT mice in the presence of Pio or in KO mice with or without Pio (data not shown). Together, these data define Pio's PPAR*γ*-dependent effects on induction of gene-specific RXR-liver DNA binding in mice on either diet. They also reveal that HFD exposure blunts the effects of Pio on such transcriptional regulation.

Next, gene set hypergeometric tests were performed on genes exhibiting Pio-induced RXR-liver DNA binding in WT mice fed either the control (Table 3A) or HFD (Table 3B). The results showed enrichment in many of the same categories identified by analyses of Pio-induced (Table 1A and B) or Pio-suppressed (Table 1C and D) hepatic gene expression. These observations imply that Pio-induced changes in RXR-liver DNA binding are transcription-inducing in some cases and transcription-suppressing in others. However, some categories enriched for genes whose expression is induced (i.e., in Table 1) were not similarly identified by these analyses of RXR-liver DNA binding (i.e., in Table 3A and B), including bile acid metabolism and cholesterol homeostasis. Presumably, this finding results from transcription-altering influences of Pio on hepatic gene expression (in Table 1A and B) that are independent of hepatocellular PPAR*γ* expression, hepatic RXR activity, or both. For example, recently described direct effects of Pio on the mitochondrial pyruvate carrier protein activity [20, 21] might have indirect transcriptional consequences. Conversely, other categories identified as enriched for RXR-bound genes in Table 3A and B were not identified by the gene set analysis of RNA-Seq data in Table 1, including glycolysis and unfolded protein response, and the significance of RXR-binding to those gene sites with respect to transcriptional regulation is uncertain. Finally, these studies did not identify any categorical gene set enrichment amongst genes with Pio-mediated suppression of RXR-liver DNA binding (Table 3C and D), nor did they demonstrate any Pio- or HFD-induced differences in such binding in the KO mice (Table 3E–H). When taken together, these data reveal a high overall degree of concordance between PPAR*γ*-dependent and Pio-induced effects on RXR-liver DNA binding to and differential hepatic expression of specific genes, and they further implicate diet as a modifier of that regulation.

### 3.4. Integrating Pio's Influences on Liver RXR-DNA Binding and Hepatic Transcription

**Figure 6:**
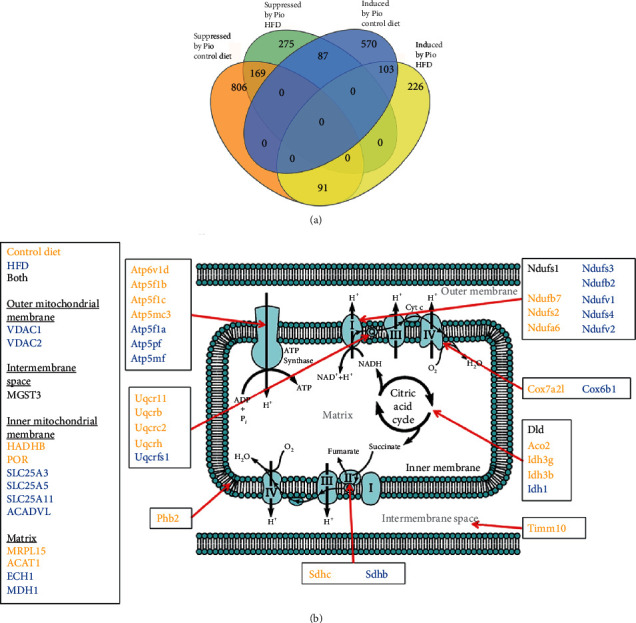
BETA Analyses of Pio's Effects on Mitochondrial Gene Expression - (a) Venn diagram analysis depicting genes identified by BETA as most likely to be regulated by Pio-induced effects on liver RXR-DNA binding in WT mice on control versus HFD. (Input RXR-liver DNA binding data from the KO mice was insufficient to execute BETA.) (b) Nuclear genes encoding mitochondrial proteins identified by BETA as likely to be Pio-induced by PPAR*γ*-dependent effects on liver RXR-DNA binding (genes in orange were identified from analyses of mice on control diet; those in blue from analyses of mice on HFD; and those in black from analyses of mice on either diet). ^∗^The mitochondria illustration is adapted from an image freely available in the public domain at https://commons.wikimedia.org/wiki/File:Mitochondrial_electron_transport_chain%E2%80%94Etc4.svg.

Finally, to further characterize and clarify the impact of diet on Pio-induced, PPAR*γ*-dependent hepatic transcriptional regulation, the RNA- and ChIP-Seq datasets generated in these studies were compared to each other using the “Binding and expression target analysis” (BETA [43],) algorithm. This analytical tool was developed to infer target gene regulation by specific transcription factors. The results here identified 760 genes bound by RXR and induced by Pio and 1066 RXR-bound genes suppressed by Pio in WT mice on control diet, and 420 induced and 531 suppressed RXR-bound gene targets of Pio in WT mice on HFD (Figure 6(a)). Most genes identified by this analysis as induced or suppressed by Pio in mice on the control diet were not similarly identified in mice on the HFD (Figure 6(a)). However, gene set over-representation analysis did demonstrate high concordance between the categorical terms identified as enriched for RXR-bound genes induced by Pio on control or HFD. Those categories include Oxidative phosphorylation, Fatty acid metabolism, and Myc targets (Table 4A and B). Further inspection of the specific Pio-regulated genes enriched in these sets revealed an abundance of nuclear genes encoding mitochondrial proteins involved in oxidative phosphorylation. Those genes include components of complexes I-IV, and the mitochondrial ATP synthase, as well as transporters, ion channels, enzymes involved in TCA cycle, and other outer membrane, intermembrane space, inner membrane, and matrix mitochondrial proteins (Figure 6(b)). Moreover, almost all of the specific mitochondrial protein-encoding genes identified in this way that were induced in mice on the control diet were not induced in mice on the HFD, and, conversely, most of those induced in HFD-treated mice were not similarly upregulated in mice on the control diet. Thus, this analysis also demonstrates that diet induces unique changes in Pio's specific transcriptional effects on nuclear genes encoding mitochondrial proteins. Gene set over-representation analysis on RXR-bound genes suppressed by Pio in WT mice was also conducted, with the results showing no overlap between functional categories enriched in suppressed genes from mice on control versus those on HFD. For example, genes associated with Unfolded protein response were enriched amongst those suppressed by Pio in WT mice on the control diet but not the HFD (Table 4C and D). These results provide further evidence for the modifying influence of diet on Pio's hepatic transcriptional effects. Finally, the differences in RXR liver DNA binding detected in the KO mice were insufficient to permit the corresponding BETA analysis on these data (Figure 5(g) and 5(h)).

## 4. Discussion

Previously published studies have reported PPAR*γ*-dependent effects of TZDs on the hepatic expression of individual target genes of interest by comparing the hepatic expression of those specific genes in WT versus PPAR*γ*-overexpressing or liver-specific PPAR*γ* KO mice [10, 12, 13, 24]. Such studies, which were recently reviewed [44], show that hepatic PPAR*γ* expression regulates the expression of adipogenic genes in the liver and also modulates extrahepatic lipid accumulation and insulin sensitivity. Nevertheless, the studies reported here are the first to comprehensively characterize such transcriptional regulation (by RNA-Seq), compare the results to genome-wide assessment of PPAR*γ*-dependent, Pio-induced effects on liver RXR-DNA binding (with ChIP-Seq), and integrate those datasets (with BETA). The results show (i) Pio's liver-PPAR*γ*-dependent hepatic transcriptional effects are highly concordant with its effects on liver RXR-DNA binding in WT mice on either diet. (ii) HFD modifies Pio's influence on such transcriptional regulation. (iii) Pio has a much broader influence on PPAR*γ*-dependent hepatic expression of nuclear genes encoding mitochondrial proteins than has previously been reported. These data provide new insights into the molecular and cellular mechanisms by which Pio and hepatocellular PPAR*γ* interact to influence hepatic gene expression. They also raise the possibility that diet is a modifier of Pio's efficacy in human NAFLD. Such efficacy was variable amongst subjects enrolled in the PIVENS trial [9]. Consistent with previously published work [10–13], our data also show that (i) hepatocellular PPAR*γ* overexpression induces hepatic steatosis. (ii) Liver-specific PPAR*γ* disruption protects mice from HFD-induced NAFLD. (iii) TZD administration (Pio here) stimulates hepatic fat accumulation in WT but not liver-specific PPAR*γ*-KO mice. Other studies have also shown that extrahepatic tissue-specific (i.e. muscle- [14] or adipocyte- [15]) PPAR*γ* deletion promotes NAFLD and that TZD-treatment exacerbates fatty liver disease in muscle-specific PPAR*γ* KO mice [14]. Taken together, our data and these considerations are also consistent with the possibility that variable responses to Pio in human NAFLD might be determined, at least in part, by the balance between TZD effects on hepatic versus extrahepatic (i.e., adipose or perhaps skeletal muscle) PPAR*γ* activity.

**Figure 7:**
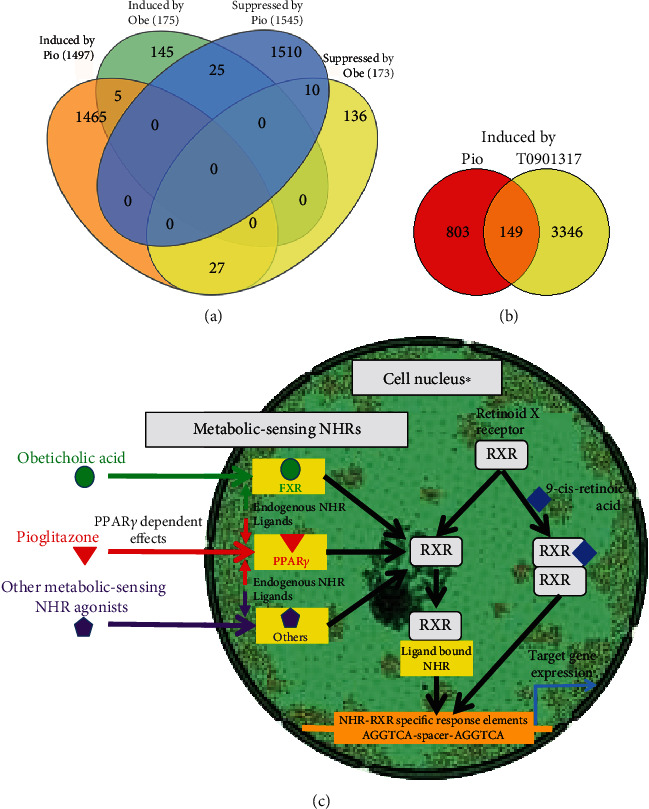
Distinct effects of Pio vs other NHR agonists. (a, b) Venn diagrams depicting (a) distinct effects of PIO (reported here) versus obeticholic acid (Obe, reported in [45]) on the hepatic transcriptome in mice and (b) PIO (reported here) versus the LXR*α* agonist T0901317 (reported in [35]) on the liver RXR cistrome. (c) Hypothetical model illustrating how different NHR-binding drugs might exert unique effects on transcriptional regulation by competing for RXR-binding, with different NHR-RXR heterodimers displaying distinct affinities for specific NHR-RXR DNA response elements based on spacer length and other factors (e.g., RXR-ligand binding by 9-cis-retinoic acid). RXR homo-dimerization also affects transcription. ^∗^The cell nucleus illustration is adapted from vectors from http://www.servier.com and licensed under the Creative Commons Attributions Unported license.

The studies here are the first to compare Pio-induced changes in the liver RXR cistrome and hepatic transcriptome with the hepatocellular PPAR*γ* dependence of those changes. Nevertheless, similar analyses of other RXR-binding, metabolic-sensing nuclear hormone receptors (NHRs) have been reported. For example, the effects of the farnesoid X receptor (FXR) agonist obeticholic acid on FXR-DNA binding and gene expression in human and mouse liver tissue were recently reported [45]. The availability of that published data permitted us to compare and contrast genes whose expression was significantly induced or suppressed by obeticholic acid in that study to those identified here as regulated by Pio in WT mouse liver. The results show distinct hepatic transcriptional effects of the FXR agonist (obeticholic acid) in that study versus those of the PPAR*γ* agonist (Pio) studied here (Figure 7(a)). Another recent study reported the RXR-liver DNA binding effects of a liver X receptor (LXR) agonist (i.e., T0901317 [35]). We also compared those published data to our characterization of Pio-induced gene-specific RXR-liver DNA binding here. That analysis also shows very little overlap between the T0901317- and Pio-induced RXR-DNA binding signatures (Figure 7(b)). These results are consistent with the idea that specific pharmacological agonists of different metabolic-sensing NHRs (e.g., Pio/PPAR*γ*, obeticholic acid/FXR, and T0901317/LXR) induce distinct effects on RXR-liver DNA binding and hepatic transcriptional regulation. That conclusion raises the possibility, also implicated by other studies [35, 46–48], that “cross-talk” in RXR-binding to different NHRs is an important determinant of NHR-ligand effects on gene expression patterns. These considerations are also consistent with the idea that drugs with distinct NHR-binding activities compete with each other and endogenous NHR ligands to exert unique effects on RXR-dependent transcriptional regulation. That concept, illustrated by the hypothetical model in Figure 7(c), raises the additional consideration that specific combinations of different NHR agonist drugs with additive beneficial (or, conversely, deleterious) effects on experimental (and perhaps human) NAFLD can be discovered using the approaches described here [49]. For example, obeticholic acid, like Pio, was also recently reported to have efficacy in human NAFLD [50]. Thus, future assessments of the combinatorial effects of Pio and obeticholic acid on transcriptional regulation and liver disease in experimental NAFLD models might inform consideration of new NHR-agonist combination intervention trials for this increasingly prevalent disease.

Pio has previously been reported to improve hepatic mitochondrial dysfunction in an in vivo NAFLD mouse model [22]. Although its effects on hepatic mitochondrial protein expression were not examined in that study, other investigations have shown that Pio stimulates mitochondrial biogenesis by upregulating specific PGC1*α*-regulated target gene expression in extrahepatic tissues [51–53]. Those observations, together with the data reported here showing that Pio broadly induces liver PPAR*γ*-dependent RXR binding and hepatic expression of many nuclear genes encoding mitochondrial proteins (Figure 6(b)), implicate such regulation as a plausible mechanism by which Pio exerts its beneficial metabolic effects. However, older studies have paradoxically reported that Pio and other TZDs inhibit hepatocellular mitochondrial function in vitro [54–56]. Thus, further investigations are required.

One limitation of the studies reported here is that the HFD regimen employed here (60% of calories from fat) had relatively modest metabolic effects. Although hepatic steatosis was not significantly increased by this regimen, the HFD-fed mice studied here did gain more weight over the experimental timecourse and display higher adiposity and serum FFA levels at the experimental endpoint, compared to mice fed the control diet (Figures 1 and 2). Reports in the literature describing results with this model are consistent with the data reported here and also note the correlation between duration-of-HFD-exposure and development of steatosis. Despite this limitation, our data nevertheless demonstrate a PPAR*γ*-dependent modifying influence of this diet on Pio-regulated changes in hepatic gene expression, including Pio's effects on mitochondrial gene expression (Figure 6(b)). Moreover, our data also indicate that the HFD regimen used here does not detectably alter liver RXR-DNA binding patterns, suggesting that the HFD effects on Pio-regulated hepatic gene expression reported here are either independent of such RXR-DNA binding or dependent on extrahepatic effects of HFD, Pio, or both (as additionally considered below). Future analyses comparing Pio's transcriptional effects here to those in mice given a “Western” or “methionine-choline deficient” diet, each of which induces more severe NAFLD phenotypes, might offer additional insights about these consideration. A second limitation is that only male mice were studied here. This strategy emerged from feasibility constraints while optimizing the experimental and analytical methodologies required for these experiments. Of note, a recent study reported sexually dimorphic RXR-liver DNA binding and hepatic expression of specific metabolic genes [36]. Thus, with the feasibility of the approach employed here now established, future investigations should investigate sex-specific effects on Pio-induced, PPAR*γ*-dependent hepatic transcriptional regulation and the influence of diet on such control. The results could have important implication for future human clinical trials testing Pio or other NHR-binding drugs. A third caveat relates to the considerations that these studies were limited to examination of hepatic transcriptional regulation, despite well-known extra-hepatic PPAR*γ*-dependent (and independent) transcriptional effects of Pio. Feasibility considerations also influenced this approach. Nevertheless, inclusion of assessments of liver RXR-DNA binding and analyses of liver-specific PPAR*γ* KO mice, here, has novel implications for consideration of direct versus indirect hepatic effects of Pio. For example, our observation that Pio does not alter liver RXR-DNA binding in liver-specific PPAR*γ* KO mice leads us to conclude not only that Pio-induced changes in RXR-liver DNA binding depend on hepatocellular PPAR*γ* expression but also that Pio-induced hepatic transcriptional effects in KO mice result, at least in part, from regulation by extrahepatic signals. Nevertheless, future studies could query relationships between and the tissue-specific dependence of Pio's hepatic and extrahepatic transcriptional effects by similar analyses of muscle and adipose collected from the liver-specific PPAR*γ* KO mice studied here and of mice with extra-hepatic PPAR*γ* disruption. Hepatic mitochondrial activity could also be assessed to evaluate the relationship between mitochondrial gene transcriptional regulation and function in these models.

In conclusion, these analyses define Pio's direct and indirect effects on hepatic transcriptional regulation and implicate diet as a modifier of those effects. They also show that Pio broadly induces liver PPAR*γ*-dependent hepatic expression of nuclear genes encoding mitochondrial proteins and that diet also impacts that regulation. Of note, only c.a. half of the subjects in the PIVENS trial showed drug-induced improvements in NAFLD [9]. Our data raise the provocative possibility that dietary effects on Pio-induced, liver PPAR*γ*-dependent hepatic expression of nuclear genes encoding mitochondrial proteins might correlate with or even mediate the variable efficacy of Pio in that trial. This consideration is important because of the current epidemic of NAFLD together with the paucity of FDA-approved drugs for treatment of that condition. Indeed, based on those circumstances, the American Association for the Study of Liver Disease (AASLD) and the European Association for the Study of the Liver (EASL) have recommended that Pio be considered in patients with biopsy-proven NASH taking into account the risks [57]. Finally, mitochondrial gene expression and function are also known to be dysregulated in other human diseases [58–60]. Thus, the data reported here might ultimately have broader relevance for future efforts to develop NHR-based therapies for human diseases.

## Figures and Tables

**Table 1:** Gene set analysis^∗^ on RNA-Seq data from WT Pio-treated vs. Pio-untreated mice on control or HFD (as designated).

Gene set name (# in Hallmark set)	Description	Overlap	*q* value
*(a) Induced by Pio in WT mice on control diet*			
FATTY_ACID_METABOLISM [158]	Genes encoding proteins involved in metabolism of fatty acids.	51	4.84*e*^−33^
ADIPOGENESIS [200]	Genes upregulated during adipocyte differentiation (adipogenesis).	48	6.52*e*^−25^
BILE_ACID_METABOLISM [112]	Genes involve in metabolism of bile acids and salts.	30	3.36*e*^−17^
CHOLESTEROL_HOMEOSTASIS [74]	Genes involved in cholesterol homeostasis.	22	7.81*e*^−14^
OXIDATIVE_PHOSPHORYLATION [200]	Genes encoding proteins involved in oxidative phosphorylation.	32	9.78*e*^−12^
PEROXISOME [104]	Genes encoding components of peroxisome.	21	7.38*e*^−10^
UV_RESPONSE_DN [144]	Genes down-regulated in response to ultraviolet (UV) radiation.	24	1.98*e*^−9^
MYC_TARGETS_V1 [200]	A subgroup of genes regulated by MYC—version 1 (v1).	28	3.84*e*^−9^
INTERFERON_GAMMA_RESPONSE [200]	Genes upregulated in response to IFNG (GeneID=3458).	26	6.74*e*^−8^
*(b) Induced by Pio in WT mice on HFD*			
FATTY_ACID_METABOLISM [158]	Genes encoding proteins involved in metabolism of fatty acids.	32	1.32*e*^−28^
OXIDATIVE_PHOSPHORYLATION [200]	Genes encoding proteins involved in oxidative phosphorylation.	33	9.38*e*^−27^
ADIPOGENESIS [200]	Genes upregulated during adipocyte differentiation (adipogenesis).	32	1.13*e*^−25^
PEROXISOME [104]	Genes encoding components of peroxisome.	20	7.76*e*^−18^
BILE_ACID_METABOLISM [112]	Genes involve in metabolism of bile acids and salts.	14	5.33*e*^−10^
GLYCOLYSIS [200]	Genes encoding proteins involved in glycolysis & gluconeogenesis.	14	8.69*e*^−7^
*(c) Suppressed by Pio in WT mice on control diet*			
UV_RESPONSE_UP [158]	Genes upregulated in response to UV radiation.	35	9.3*e*^−16^
HEME_METABOLISM [200]	Genes involved in metabolism of heme & erythroblast differentiation.	32	1.43*e*^−10^
MTORC1_SIGNALING [200]	Genes upregulated through activation of mTORC1 complex.	28	3.4*e*^−8^
P53_PATHWAY [200]	Genes involved in p53 pathways and networks.	28	3.4*e*^−8^
APOPTOSIS [161]	Genes mediating apoptosis by activation of caspases.	24	8.69*e*^−8^
HYPOXIA [200]	Genes upregulated in response to low oxygen levels.	27	8.69*e*^−8^
DNA_REPAIR [150]	Genes involved in DNA repair.	23	8.69*e*^−8^
XENOBIOTIC_METABOLISM [200]	Genes encoding proteins that process drugs & other xenobiotics.	26	2.71*e*^−7^
UNFOLDED_PROTEIN_RESPONSE [113]	Genes upregulated during unfolded protein response.	19	2.71*e*^−7^
MYC_TARGETS_V1 [200]	A subgroup of genes regulated by MYC - version 1 (v1).	25	9.04*e*^−7^
*(d) Suppressed by Pio in WT mice on HFD*			
No overlaps found			

^∗^Using the Broad Institute platform with the Hallmark gene sets platform to identify the top 10 categories with *p* ≤ 1*e*^−6^ (see text for details).

**Table 2:** Gene set analysis^∗^ on RNA-Seq data from Pio-treated vs. Pio-untreated KO mice on control or HFD (as designated).

Gene set name (# in Hallmark set)	Description	Overlap	*q* value
*(a) Induced by Pio in KO mice on control diet*			
CHOLESTEROL_HOMEOSTASIS [74]	Genes involved in cholesterol homeostasis.	20	1.04*e*^−18^
ESTROGEN_RESPONSE_LATE [200]	Genes defining late response to estrogen.	22	3.64*e*^−12^
TNFA_SIGNALING_VIA_NFKB [200]	Genes regulated by NF-*κ*B in response to TNF (GeneID=7124).	21	2.17*e*^−11^
MTORC1_SIGNALING [200]	Genes upregulated through activation of mTORC1 complex.	20	1.38*e*^−10^
APOPTOSIS [161]	Genes mediating apoptosis by activation of caspases.	18	1.83*e*^−10^
ESTROGEN_RESPONSE_EARLY [200]	Genes defining early response to estrogen.	19	7.36*e*^−10^
NOTCH_SIGNALING [32]	Genes upregulated by activation of notch signaling.	8	8.45*e*^−8^
HYPOXIA [200]	Genes upregulated in response to low oxygen levels (hypoxia).	16	1.98*e*^−7^
UV_RESPONSE_UP [158]	Genes upregulated in response to ultraviolet (UV) radiation.	14	3.57*e*^−7^
ADIPOGENESIS [200]	Genes upregulated during adipocyte differentiation (adipogenesis).	15	9.91*e*^−7^
*(b) Induced by Pio in KO mice on HFD*			
No significantly differentially expressed genes			
*(c) Suppressed by Pio in KO mice on control diet*			
XENOBIOTIC_METABOLISM [200]	Genes encoding proteins that process drugs and xenobiotics.	24	2.13*e*^−13^
*(d) Suppressed by Pio in KO mice on HFD*			
No significantly differentially expressed genes			

^∗^Using the Broad Institute platform with the Hallmark gene set platform to identify the top 10 categories with *p* ≤ 1*e*^−6^ (see text for details).

**Table 3:** Gene set analysis^∗^ on RXR ChIP-Seq data from Pio-treated vs. Pio-untreated WT or KO mice on control or HFD (as designated).

Gene set name (# in Hallmark set)	Description	Overlap	*q* value
*(a) Induced by Pio in WT mice on control diet*			
OXIDATIVE_PHOSPHORYLATION [200]	Genes encoding proteins involved in oxidative phosphorylation.	48	2.55*e*^−35^
MYC_TARGETS_V1 [200]	A subgroup of genes regulated by MYC - version 1 (v1).	40	2.33*e*^−26^
ADIPOGENESIS [200]	Genes upregulated during adipocyte differentiation (adipogenesis).	31	3.41*e*^−17^
ESTROGEN_RESPONSE_LATE [200]	Genes defining late response to estrogen.	24	5.47*e*^−11^
FATTY_ACID_METABOLISM [158]	Genes encoding proteins involved in metabolism of fatty acids.	21	1.34*e*^−10^
MTORC1_SIGNALING [200]	Genes upregulated through activation of mTORC1 complex.	23	2.45*e*^−10^
GLYCOLYSIS [200]	Genes encoding proteins involved in glycolysis and gluconeogenesis.	21	8.17*e*^−9^
UNFOLDED_PROTEIN_RESPONSE [113]	Genes upregulated during unfolded protein response.	16	8.78*e*^−9^
ESTROGEN_RESPONSE_EARLY [200]	Genes defining early response to estrogen.	19	2*e*^−7^
DNA_REPAIR [150]	Genes involved in DNA repair.	16	4.31*e*^−7^
*(b) Induced by Pio in WT mice on HFD*			
OXIDATIVE_PHOSPHORYLATION [200]	Genes encoding proteins involved in oxidative phosphorylation.	31	2.38*e*^−24^
MYC_TARGETS_V1 [200]	A subgroup of genes regulated by MYC—version 1 (v1).	25	1.66*e*^−17^
ADIPOGENESIS [200]	Genes upregulated during adipocyte differentiation (adipogenesis).	20	2.72*e*^−12^
ESTROGEN_RESPONSE_LATE [200]	Genes defining late response to estrogen.	15	1.22*e*^−7^
*(c, d) Suppressed by Pio in WT mice on control or HFD*			
No overlaps found.			
*(e, f, g, h) Induced or suppressed by Pio or HFD in KO mice in presence or absence of HFD or Pio*			
No overlaps found.			

^∗^Using the Broad Institute platform with the Hallmark gene set platform to identify the top 10 categories with *p* ≤ 1*e*^−6^ (see text for details).

**Table 4:** Gene set analysis^∗^ on BETA data from WT Pio-treated vs. Pio-untreated mice on control or HFD (as designated).

Gene set name (# in Hallmark set)	Description	Overlap	*q* value
*(a) Induced by Pio in WT mice on control diet*			
OXIDATIVE_PHOSPHORYLATION [200]	Genes encoding proteins involved in oxidative phosphorylation.	28	7.09*e*^−16^
FATTY_ACID_METABOLISM [158]	Genes encoding proteins involved in metabolism of fatty acids.	25	9.9*e*^−16^
ADIPOGENESIS [200]	Genes upregulated during adipocyte differentiation (adipogenesis).	20	5.16*e*^−9^
MYC_TARGETS_V1 [200]	A subgroup of genes regulated by MYC—version 1 (v1).	19	2.54*e*^−8^
*(b) Induced by Pio in WT mice on HFD*			
OXIDATIVE_PHOSPHORYLATION [200]	Genes encoding proteins involved in oxidative phosphorylation.	23	5.46*e*^−16^
MYC_TARGETS_V1 [200]	A subgroup of genes regulated by MYC - version 1 (v1).	15	5.89*e*^−8^
FATTY_ACID_METABOLISM [158]	Genes encoding proteins involved in metabolism of fatty acids.	13	1.52*e*^−7^
*(c) Suppressed by Pio in WT mice on control diet*			
UV_RESPONSE_UP [158]	Genes upregulated in response to ultraviolet (UV) radiation.	23	1.49*e*^−9^
UNFOLDED_PROTEIN_RESPONSE [113]	Genes upregulated during unfolded protein response, a cellular stress response related to the endoplasmic reticulum.	17	1.66*e*^−7^
E2F_TARGETS [200]	Genes encoding cell cycle related targets of E2F transcription factors.	21	8.05*e*^−7^
P53_PATHWAY [200]	Genes involved in p53 pathways and networks.	21	8.05*e*^−7^
TNFA_SIGNALING_VIA_NFKB [200]	Genes regulated by NF-kB in response to TNF (GeneID=7124).	21	8.05*e*^−7^
*(d) Suppressed by Pio in WT mice on HFD*			
MYC_TARGETS_V1 [200]	A subgroup of genes regulated by MYC—version 1 (v1).	17	4.45 e^−8^

^∗^Using the Broad Institute platform with the Hallmark gene set platform to identify the top 10 categories with *p* ≤ 1*e*^−6^ (see text for details).

## Data Availability

The ChIP- and RNA-Seq data used to support the findings of the studies reported here have been deposited in NCBI's Gene Expression Omnibus (GEO [34]) and will be made accessible to the public as GEO series records GSE137820 (for ChIP-Seq data) and GSE140607 (for RNA-Seq data) at the time this manuscript is accepted for publication. In addition, excel spreadsheets summarizing the results of the RNA-Seq, RXR-ChIP, and BETA data analyses (i.e., for RNA-Seq, lists of differentially expressed genes with fold change, confidence interval, *p* and *q* value; for ChIP-Seq, lists of differentially RXR-bound genes, with fold difference, *q* value, and genomic site of binding; for BETA, gene name, rank, and rank product) are provided in Supplementary Table S2.

## Abbreviations

ChIP-Seq: Chromatin immunoprecipitation with next generation sequencing;
ENCODE: Encyclopedia of DNA elements
FXR: Farnesoid X receptor
GSEA: Gene set enrichment analysis
HFD: High-fat diet
KO: Knockout
LXR: Liver X receptor
NAFLD: Nonalcoholic fatty liver disease
NHR: Nuclear hormone receptor
ns: Not significant
PCA: Principle component analysis
PIO: Pioglitazone
PIVENS: Pioglitazone versus Vitamin E versus Placebo for the Treatment of Non-Diabetic Patients with Nonalcoholic Steatohepatitis
PPAR: Peroxisome proliferator receptor
PPRE: Peroxisome proliferator receptor response element
RNA-Seq: RNA sequencing with next generation sequencing
RXR: Retinoid X receptor
TZD: Thiazolidinedione
UPR: Unfolded protein response
UTR: Untranslated region
WT: Wildtype.

## Supplementary Materials

Supplementary Materials Supplementary Figure S1: immunoblot analysis of the anti-RXR*α*/*β*/*γ* (Santa Cruz sc-774) antibody that was used for the ChIP-Seq studies reported here. The predominant detectable band in all liver samples (i.e., from control, Pio-treated, HFD-treated, and HFD-Pio-treated mice) is ~53 kDa band, which is the molecular weight of RXR, and accounts for >50% of the total detected signal in each lane (average 81% across all samples, as assessed using Odyssey Infrared Imaging System Application Software Version 3.0.21, Licor Biosciences), which is consistent with the ENCODE and modENCODE guidelines [37, 61]. Supplementary Figure S2: the left panel shows initial body weights (in grams) of WT and KO mice (as designated) that were subsequently assigned to the indicated experimental groups (no significant differences between groups). The right panel shows a summary of initial body weights in all WT versus all KO mice (*p* < 0.05). Supplementary Figure S3: summary of RT-qPCR based analyses of exemplar genes whose RNA-Seq based patterns of expression are depicted in Figure 3. Supplementary Table S1: gene-specific oligonucleotide primers for RT-qPCR. Supplementary Table S2: RNA-Seq, ChIP-Seq, and BETA Analyses Results—see excel Table S2. This excel spreadsheet contains (A) List of Experimental Groups, (B) a merged summary of all RNA-Seq differential expression analyses, summaries of differentially RXR-bound peaks identified by ChIP-Seq analyses of liver samples from wildtype (C) Pio vs. control or (D) HFD-Pio-treated vs. HFD-treated mice, and (E) summary lists from BETA analyses of RNA- and ChIP-Seq data from comparisons of Pio vs. control and HFD-Pio vs. HFD (including rank, Gene name, and rank product). Supplementary Table S3: GAGE results—see excel Table S3: this excel spreadsheet contains GAGE gene set expression perturbation test results for all comparisons between groups conducted by Limma. Log 2 fold changes of all genes within specific gene sets were compared to all those outside those sets using Student's *t*-tests. Results are reported as mean log 2 fold-change, *p* value, and Benjamini-Hochberg FDR adjusted *p* value. Supplementary Table S4: gene set analysis on RNA-Seq data from HFD vs. control diet-treated WT mice: these tables were generated using the Broad Institute platform with the Hallmark gene set platform to identify the top 10 categories with *p* ≤ 1*e*^−6^ (see text for additional details). Supplementary Table S5: gene set analysis^∗^ on RNA-Seq data from HFD vs. control diet-treated KO mice: these tables were generated using the Broad Institute platform with the Hallmark gene set platform to identify the top 10 categories with *p* ≤ 1*e*^−6^ (see text for additional details).
