# Supplementary material for: Diet Modifies Pioglitazone's Influence on Hepatic PPARγ-Regulated Mitochondrial Gene Expression
Source: PPAR Res. 2020 Sep 10;2020:3817573. doi: 10.1155/2020/3817573 (PMC7501566; doi:10.1155/2020/3817573)
Supplement: Supplementary Materials — Supplementary Figure S1: immunoblot analysis of the anti-RXRα/β/γ (Santa Cruz sc-774) antibody that was used for the ChIP-Seq studies reported here. The predominant detectable band in all liver samples (i.e., from control, Pio-treated, HFD-treated, and HFD-Pio-treated mice) is ~53 kDa band, which is the molecular weight of RXR, and accounts for >50% of the total detected signal in each lane (average 81% across all samples, as assessed using Odyssey Infrared Imaging System Application Software Version 3.0.21, Licor Biosciences), which is consistent with the ENCODE and modENCODE guidelines [37, 61]. Supplementary Figure S2: the left panel shows initial body weights (in grams) of WT and KO mice (as designated) that were subsequently assigned to the indicated experimental groups (no significant differences between groups). The right panel shows a summary of initial body weights in all WT versus all KO mice (p < 0.05). Supplementary Figure S3: summary of RT-qPCR based analyses of exemplar genes whose RNA-Seq based patterns of expression are depicted in Figure 3. Supplementary Table S1: gene-specific oligonucleotide primers for RT-qPCR. Supplementary Table S2: RNA-Seq, ChIP-Seq, and BETA Analyses Results—see excel Table S2. This excel spreadsheet contains (A) List of Experimental Groups, (B) a merged summary of all RNA-Seq differential expression analyses, summaries of differentially RXR-bound peaks identified by ChIP-Seq analyses of liver samples from wildtype (C) Pio vs. control or (D) HFD-Pio-treated vs. HFD-treated mice, and (E) summary lists from BETA analyses of RNA- and ChIP-Seq data from comparisons of Pio vs. control and HFD-Pio vs. HFD (including rank, Gene name, and rank product). Supplementary Table S3: GAGE results—see excel Table S3: this excel spreadsheet contains GAGE gene set expression perturbation test results for all comparisons between groups conducted by Limma. Log 2 fold changes of all genes within specific gene sets were compared to all those outsi [file 3817573.f1.zip › 20200818 PPAR Research Supporting Info Fig S1-S3 Tables S1, S4-S5 FINAL SUBMITTED.docx]

**Diet modifies pioglitazone’s influence on hepatic PPARγ-regulated mitochondrial gene expression**

Sakil Kulkarni^1^, Jiansheng Huang^1^, Eric Tycksen^2^, Paul F. Cliften^2^, and David A. Rudnick^1,3,*^

**Supporting Information – List of Supporting Materials**

RXR ChIP-Seq: Additional considerations regarding ENCODE quality control guidelines (see below).

Supplementary Figure S1: Immunoblot of anti-RXR ChIP-Seq antibody reactivity with liver (see below)

Supplementary Figure S2: Initial mouse body weights (see below)

Supplementary Figure S3: Exemplar gene expression by RT-qPCR (see below)

1. Supplementary Table S1: Gene-Specific Oligonucleotide Primers for RT-qPCR (see below)

2. Supplementary Table S2: RNA-Seq, RXR-ChIP Seq, and BETA data summary (see Excel Table S2)

3. Supplementary Table S3 GAGE analysis for gene-set level changes (See Excel Table S3)

4. Supplementary Table S4: GSEA on RNA-Seq data from HFD vs. control diet in WT mice

5. Supplementary Table S5: GSEA on RNA-Seq data from HFD vs. control diet in KO mice

**RXR ChIP-Seq: Additional considerations regarding ENCODE quality control guidelines.** The studies reported here adhered to the guidelines and practices recommended for analyses and quality control of ChIP-Seq data by the Encyclopedia of DNA elements (i.e. ENCODE and modENCODE) consortia guidelines (1). These include recommendations to validate the specificity of the ChIP target transcription factor antibody by immunoblot and quantification, as shown in Supplementary Figure 1, and also to assess each replicate of immunoprecipitated chromatin for quality metrics including NSC, RSC, Qtag score, and Irreproducible Discovery Rate (IDR) data. The latter analyses showed that the RSC scores for our ChIP samples were consistently lower than the ENCODE guidelines, which has also previously been reported in ChIP-seq samples prepared from liver tissue versus other sources (as shown in Table S1 in reference (2)). IDR analyses on our ChiP-seq samples were also conducted and showed variability above the self-consistency thresholds within the WT treatment groups. However, comparisons of the maximum number of peaks from the original replicates to the pooled pseudo-replicate peak number were within the ENCODE guidelines. Also of note, our biological replicates were from separate animals rather than from independent cell cultures, embryo pools or tissue samples. The latter biological samples account for most substrates of the ENCODE experiments. Thus, we anticipated some variability when considering experimental design, based on which we used liver samples from each of 5 mouse replicates per group, retained all replicates for these analyses, and applied additional stringencies, as described below, for those analyses.

Efforts to conduct PPARγ ChIP-Seq analyses on liver were also attempted here, based on similar analyses of other cell types and tissues (e.g. adipose, adipocyte lineage cells, HT-29, and human leukemia cells) available in GEO DataSets (<https://www.ncbi.nlm.nih.gov/gds/>). However, these efforts were unsuccessful because, despite testing multiple ChIP-grade antibodies used to generate ChIP data from those other cell types and tissues (as reported in GEO DataSets) we were unable to identify a PPARγ antibody meeting the ENCODE guidelines described above when tested on mouse liver (S. Kulkarni, J. Huang, and D.A. Rudnick, unpublished observations). We were also unable to identify any reports describing PPARγ ChIP-Seq analyses of liver tissue either in PubMed or GEO Datasets. Based on these considerations and in order to assess the liver PPARγ-dependence of RXR liver DNA binding in the samples studied here, RXR-ChIP-Seq studies were conducted on livers from WT and liver-specific PPARγ KO mice and the result compared.

**Supplementary Figure S1: Immunoblot analysis of anti-RXR ChIP-Seq antibody reactivity with liver**

**
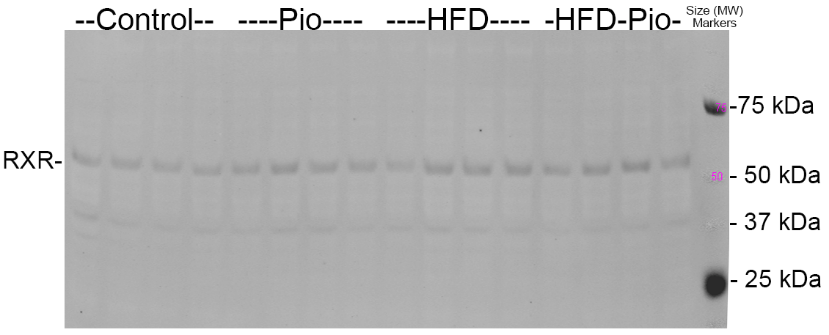
Supplementary Figure 1:** Immunoblot analysis of the anti-RXRα/β/γ (Santa Cruz sc-774) antibody that was used for the ChIP-Seq studies reported here. The predominant detectable band in all liver samples (i.e. from control, Pio-treated, HFD-treated and HFD-Pio-treated mice) is ~53 kDa band, which is the molecular weight of RXR, and accounts for >50% of the total detected signal in each lane (average 81% across all samples, as assessed using Odyssey Infrared Imaging System Application Software Version 3.0.21, Licor Biosciences), which is consistent with the ENCODE and modENCODE guidelines (1,2).

**Supplementary Figure S2: Initial mouse body weights**


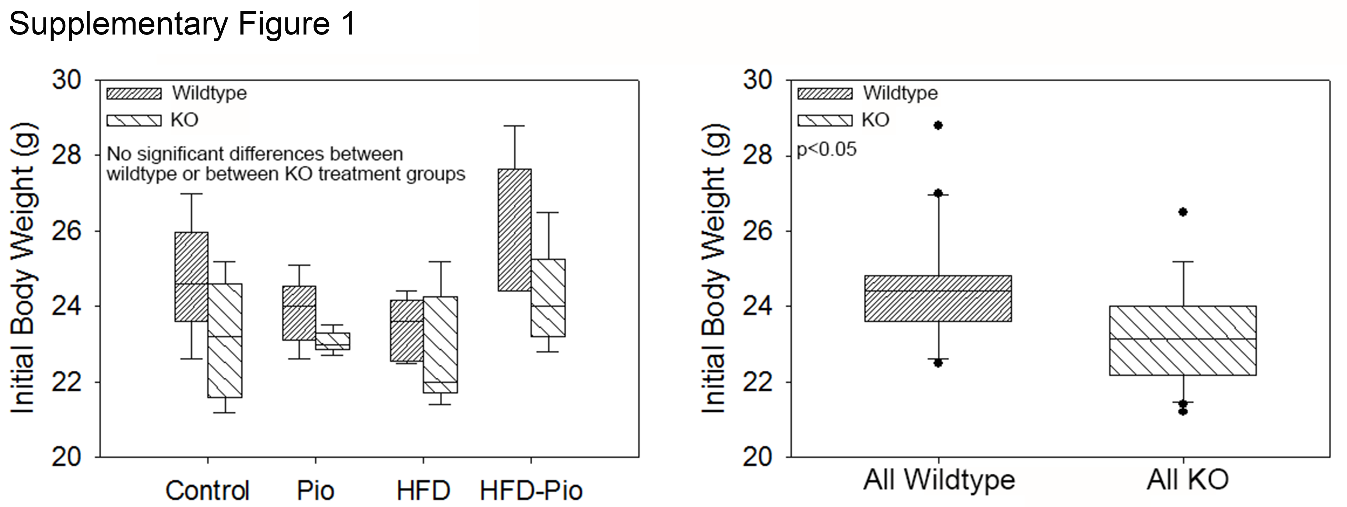


**Supplementary Figure S2:** The left panel shows initial body weights (in grams) of WT and KO mice (as designated) that were subsequently assigned to the indicated experimental groups (no significant differences between groups). The right panel shows a summary of initial body weights in all WT versus all KO mice (p<0.05).

**Supplementary Figure S3: RT-qPCR validation of patterns of exemplar gene expression in Figure 3**


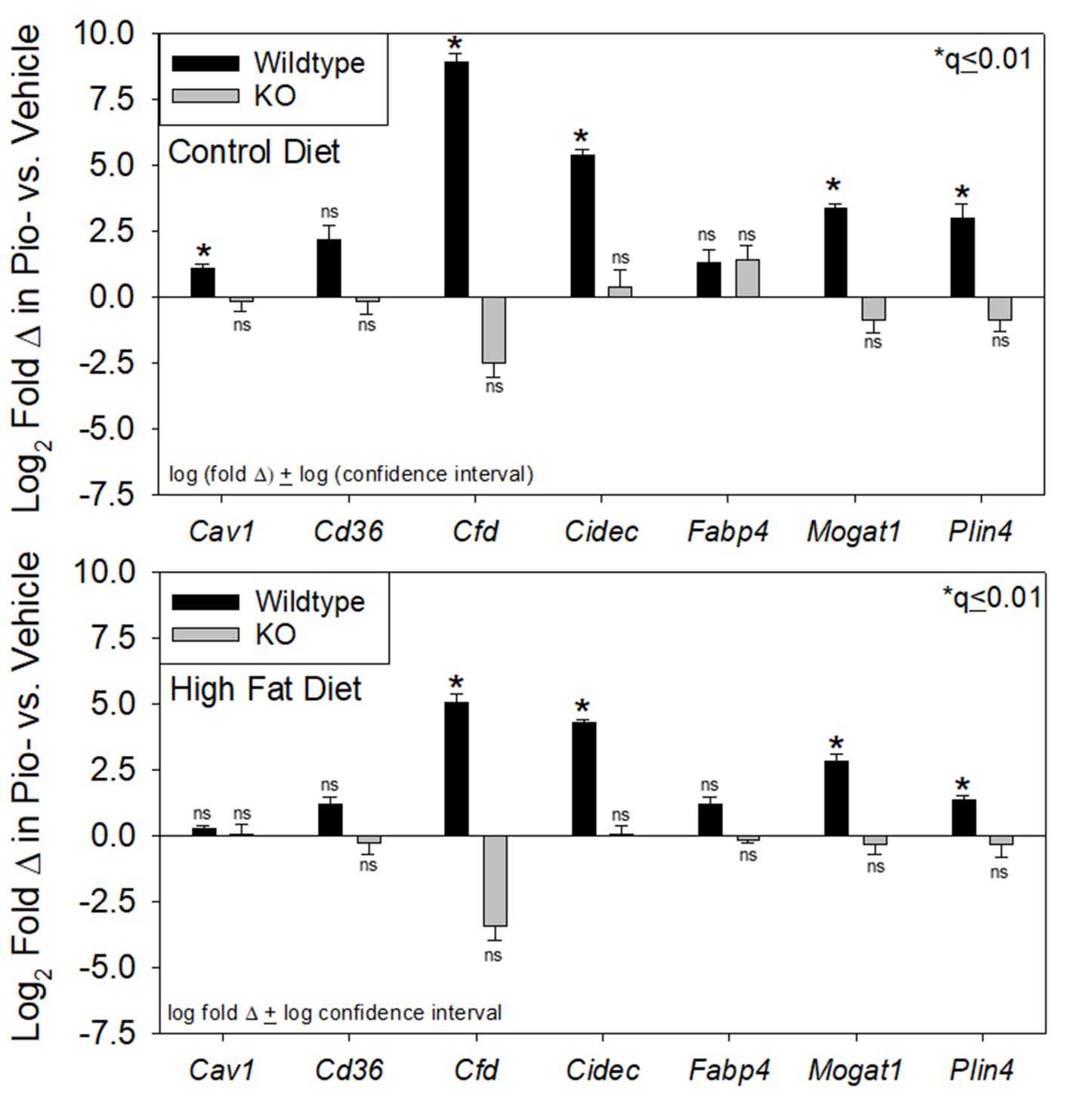


**Supplementary Figure S3:** Summary of RT-qPCR based analyses of exemplar genes whose RNA-Seq based patterns of expression are depicted in Figure 3.

**Supplementary Table S1: Gene-Specific Oligonucleotide Primers for RT-qPCR:**

| **Gene** | **Forward Primer** | **Reverse Primer** |
| --- | --- | --- |
| Cav1 | GCGCACACCAAGGAGATTGA | GATGCCGTCGAAACTGTGTG |
| Cd36 | TGGTGCAGTCCTGGCTGTGTT | GTGTGGTCCTCGGGGTCCTG |
| Cfd | GCTGTCAGAATGCACAGCTC | CAGAATCCGGCCTCGGG |
| Cidec | GATCGGAAGGTTCGCAAAGG | TGTCTCCACGATTGTGCCAT |
| Fabp4 | GCAGCTTCCTTCTCACCTTGA | TGGCAAAGCCCACTCCTACT |
| Mogat1 | GAGTTCGCGCCACTCAACAC | GTACAGGACCAGCATCACCAT |
| Plin4 | CTGCCCCCTCATCTAAAGTGT | TCCTTGTCTCCAGCTGTCTGTTC |

**Supplementary Table S4: Gene Set Analysis* on RNA-Seq Data from HFD vs. Control diet Treated WT Mice**

**A. Induced by HFD in WT mice in the absence of Pio**

| **Gene Set Name [# in Hallmark set]** | **Description** | **Overlap** | **q-value** |
| --- | --- | --- | --- |
| [FATTY_ACID_METABOLISM](http://software.broadinstitute.org/gsea/msigdb/geneset_page.jsp?geneSetName=HALLMARK_FATTY_ACID_METABOLISM) [[158](http://software.broadinstitute.org/gsea/msigdb/download_geneset.jsp?geneSetName=HALLMARK_FATTY_ACID_METABOLISM&fileType=grp)] | Genes encoding proteins involved in metabolism of fatty acids. | 18 | 8.09 e^-19^ |
| [BILE_ACID_METABOLISM](http://software.broadinstitute.org/gsea/msigdb/geneset_page.jsp?geneSetName=HALLMARK_BILE_ACID_METABOLISM) [[112](http://software.broadinstitute.org/gsea/msigdb/download_geneset.jsp?geneSetName=HALLMARK_BILE_ACID_METABOLISM&fileType=grp)] | Genes involve in metabolism of bile acids and salts. | 10 | 1.59 e^-9^ |
| [XENOBIOTIC_METABOLISM](http://software.broadinstitute.org/gsea/msigdb/geneset_page.jsp?geneSetName=HALLMARK_XENOBIOTIC_METABOLISM) [[200](http://software.broadinstitute.org/gsea/msigdb/download_geneset.jsp?geneSetName=HALLMARK_XENOBIOTIC_METABOLISM&fileType=grp)] | Genes encoding proteins involved in processing drugs & xenobiotics. | 12 | 1.59 e^-9^ |

**B. Induced by HFD in WT mice in the presence of Pio**

| **Gene Set Name [# in Hallmark set]** | **Description** | **Overlap** | **q-value** |
| --- | --- | --- | --- |
| [FATTY_ACID_METABOLISM](http://software.broadinstitute.org/gsea/msigdb/geneset_page.jsp?geneSetName=HALLMARK_FATTY_ACID_METABOLISM) [[158](http://software.broadinstitute.org/gsea/msigdb/download_geneset.jsp?geneSetName=HALLMARK_FATTY_ACID_METABOLISM&fileType=grp)] | Genes encoding proteins involved in metabolism of fatty acids. | 56 | 2.4 e^-37^ |
| [OXIDATIVE_PHOSPHORYLATION](http://software.broadinstitute.org/gsea/msigdb/geneset_page.jsp?geneSetName=HALLMARK_OXIDATIVE_PHOSPHORYLATION)  [[200](http://software.broadinstitute.org/gsea/msigdb/download_geneset.jsp?geneSetName=HALLMARK_OXIDATIVE_PHOSPHORYLATION&fileType=grp)] | Genes encoding proteins involved in oxidative phosphorylation. | 52 | 1.79 e^-27^ |
| [PEROXISOME](http://software.broadinstitute.org/gsea/msigdb/geneset_page.jsp?geneSetName=HALLMARK_PEROXISOME) [[104](http://software.broadinstitute.org/gsea/msigdb/download_geneset.jsp?geneSetName=HALLMARK_PEROXISOME&fileType=grp)] | Genes encoding components of peroxisome. | 36 | 6.03 e^-24^ |
| [XENOBIOTIC_METABOLISM](http://software.broadinstitute.org/gsea/msigdb/geneset_page.jsp?geneSetName=HALLMARK_XENOBIOTIC_METABOLISM) [[200](http://software.broadinstitute.org/gsea/msigdb/download_geneset.jsp?geneSetName=HALLMARK_XENOBIOTIC_METABOLISM&fileType=grp)] | Genes encoding proteins involved in processing drugs & xenobiotics. | 43 | 1.59 e^-19^ |
| [ADIPOGENESIS](http://software.broadinstitute.org/gsea/msigdb/geneset_page.jsp?geneSetName=HALLMARK_ADIPOGENESIS) [[200](http://software.broadinstitute.org/gsea/msigdb/download_geneset.jsp?geneSetName=HALLMARK_ADIPOGENESIS&fileType=grp)] | Genes up-regulated during adipocyte differentiation (adipogenesis). | 37 | 8.87 e^-15^ |
| [HEME_METABOLISM](http://software.broadinstitute.org/gsea/msigdb/geneset_page.jsp?geneSetName=HALLMARK_HEME_METABOLISM) [[200](http://software.broadinstitute.org/gsea/msigdb/download_geneset.jsp?geneSetName=HALLMARK_HEME_METABOLISM&fileType=grp)] | Genes involved in metabolism of heme and erythroblast differentiation. | 37 | 8.87 e^-15^ |
| [BILE_ACID_METABOLISM](http://software.broadinstitute.org/gsea/msigdb/geneset_page.jsp?geneSetName=HALLMARK_BILE_ACID_METABOLISM) [[112](http://software.broadinstitute.org/gsea/msigdb/download_geneset.jsp?geneSetName=HALLMARK_BILE_ACID_METABOLISM&fileType=grp)] | Genes involve in metabolism of bile acids and salts. | 27 | 6.27 e^-14^ |
| [DNA_REPAIR](http://software.broadinstitute.org/gsea/msigdb/geneset_page.jsp?geneSetName=HALLMARK_DNA_REPAIR) [[150](http://software.broadinstitute.org/gsea/msigdb/download_geneset.jsp?geneSetName=HALLMARK_DNA_REPAIR&fileType=grp)] | Genes involved in DNA repair. | 30 | 3.88 e^-13^ |
| [UV_RESPONSE_UP](http://software.broadinstitute.org/gsea/msigdb/geneset_page.jsp?geneSetName=HALLMARK_UV_RESPONSE_UP) [[158](http://software.broadinstitute.org/gsea/msigdb/download_geneset.jsp?geneSetName=HALLMARK_UV_RESPONSE_UP&fileType=grp)] | Genes up-regulated in response to ultraviolet (UV) radiation. | 27 | 2.87 e^-10^ |
| [REACTIVE_OXYGEN_SPECIES_ PATHWAY](http://software.broadinstitute.org/gsea/msigdb/geneset_page.jsp?geneSetName=HALLMARK_REACTIVE_OXYGEN_SPECIES_PATHWAY) [[49](http://software.broadinstitute.org/gsea/msigdb/download_geneset.jsp?geneSetName=HALLMARK_REACTIVE_OXYGEN_SPECIES_PATHWAY&fileType=grp)] | Genes up-regulated by reactive oxygen species (ROS). | 14 | 1.1 e^-8^ |

**C. Suppressed by HFD in WT mice in the absence of Pio:** No overlaps found.

**D. Suppressed by HFD in the presence of Pio**

| **Gene Set Name [# in Hallmark set]** | **Description** | **Overlap** | **q-value** |
| --- | --- | --- | --- |
| [HALLMARK_CHOLESTEROL_ HOMEOSTASIS](http://software.broadinstitute.org/gsea/msigdb/geneset_page.jsp?geneSetName=HALLMARK_CHOLESTEROL_HOMEOSTASIS) [[74](http://software.broadinstitute.org/gsea/msigdb/download_geneset.jsp?geneSetName=HALLMARK_CHOLESTEROL_HOMEOSTASIS&fileType=grp)] | Genes involved in cholesterol homeostasis. | 27 | 4.17 e^-19^ |
| [HALLMARK_MYC_TARGETS_V1](http://software.broadinstitute.org/gsea/msigdb/geneset_page.jsp?geneSetName=HALLMARK_MYC_TARGETS_V1) [[200](http://software.broadinstitute.org/gsea/msigdb/download_geneset.jsp?geneSetName=HALLMARK_MYC_TARGETS_V1&fileType=grp)] | A subgroup of genes regulated by MYC - version 1 (v1). | 34 | 5.18 e^-13^ |
| [HALLMARK_MTORC1_SIGNALING](http://software.broadinstitute.org/gsea/msigdb/geneset_page.jsp?geneSetName=HALLMARK_MTORC1_SIGNALING)  [[200](http://software.broadinstitute.org/gsea/msigdb/download_geneset.jsp?geneSetName=HALLMARK_MTORC1_SIGNALING&fileType=grp)] | Genes up-regulated through activation of mTORC1 complex. | 33 | 2 e^-12^ |
| [HALLMARK_MITOTIC_SPINDLE](http://software.broadinstitute.org/gsea/msigdb/geneset_page.jsp?geneSetName=HALLMARK_MITOTIC_SPINDLE) [[199](http://software.broadinstitute.org/gsea/msigdb/download_geneset.jsp?geneSetName=HALLMARK_MITOTIC_SPINDLE&fileType=grp)] | Genes important for mitotic spindle assembly. | 32 | 7.29 e^-12^ |
| [HALLMARK_INTERFERON_GAMMA_ RESPONSE](http://software.broadinstitute.org/gsea/msigdb/geneset_page.jsp?geneSetName=HALLMARK_INTERFERON_GAMMA_RESPONSE) [[200](http://software.broadinstitute.org/gsea/msigdb/download_geneset.jsp?geneSetName=HALLMARK_INTERFERON_GAMMA_RESPONSE&fileType=grp)] | Genes up-regulated in response to IFNG [GeneID=3458]. | 27 | 2.06 e^-8^ |
| [HALLMARK_G2M_CHECKPOINT](http://software.broadinstitute.org/gsea/msigdb/geneset_page.jsp?geneSetName=HALLMARK_G2M_CHECKPOINT) [[200](http://software.broadinstitute.org/gsea/msigdb/download_geneset.jsp?geneSetName=HALLMARK_G2M_CHECKPOINT&fileType=grp)] | Genes involved in the G2/M checkpoint. | 26 | 7.58 e^-8^ |
| [HALLMARK_APICAL_JUNCTION](http://software.broadinstitute.org/gsea/msigdb/geneset_page.jsp?geneSetName=HALLMARK_APICAL_JUNCTION) [[200](http://software.broadinstitute.org/gsea/msigdb/download_geneset.jsp?geneSetName=HALLMARK_APICAL_JUNCTION&fileType=grp)] | Genes encoding components of apical junction complex. | 25 | 2.75 e^-7^ |
| [HALLMARK_UV_RESPONSE_DN](http://software.broadinstitute.org/gsea/msigdb/geneset_page.jsp?geneSetName=HALLMARK_UV_RESPONSE_DN) [[144](http://software.broadinstitute.org/gsea/msigdb/download_geneset.jsp?geneSetName=HALLMARK_UV_RESPONSE_DN&fileType=grp)] | Genes down-regulated in response to UV radiation. | 20 | 9.55 e^-7^ |

**Supplementary Table S5: Gene Set Analysis* on RNA-Seq Data from HFD vs. Control Diet Treated KO Mice**

**A. Induced by HFD in KO mice in the absence of Pio**

| **Gene Set Name [# in Hallmark set]** | **Description** | **Overlap** | **q-value** |
| --- | --- | --- | --- |
| [CHOLESTEROL_HOMEOSTASIS](http://software.broadinstitute.org/gsea/msigdb/geneset_page.jsp?geneSetName=HALLMARK_CHOLESTEROL_HOMEOSTASIS) [[74](http://software.broadinstitute.org/gsea/msigdb/download_geneset.jsp?geneSetName=HALLMARK_CHOLESTEROL_HOMEOSTASIS&fileType=grp)] | Genes involved in cholesterol homeostasis. | 22 | 5.56 e^-29^ |
| [MTORC1_SIGNALING](http://software.broadinstitute.org/gsea/msigdb/geneset_page.jsp?geneSetName=HALLMARK_MTORC1_SIGNALING) [[200](http://software.broadinstitute.org/gsea/msigdb/download_geneset.jsp?geneSetName=HALLMARK_MTORC1_SIGNALING&fileType=grp)] | Genes up-regulated by activation of mTORC1 complex. | 14 | 2.04 e^-9^ |

**B. Induced by HFD in KO mice in the presence of Pio**

| **Gene Set Name [# in Hallmark set]** | **Description** | **Overlap** | | **q-value** |
| --- | --- | --- | --- | --- |
| [CHOLESTEROL_HOMEOSTASIS](http://software.broadinstitute.org/gsea/msigdb/geneset_page.jsp?geneSetName=HALLMARK_CHOLESTEROL_HOMEOSTASIS) [[74](http://software.broadinstitute.org/gsea/msigdb/download_geneset.jsp?geneSetName=HALLMARK_CHOLESTEROL_HOMEOSTASIS&fileType=grp)] | Genes involved in cholesterol homeostasis. | | 11 | 1.26 e^-14^ |
| [XENOBIOTIC_METABOLISM](http://software.broadinstitute.org/gsea/msigdb/geneset_page.jsp?geneSetName=HALLMARK_XENOBIOTIC_METABOLISM) [[200](http://software.broadinstitute.org/gsea/msigdb/download_geneset.jsp?geneSetName=HALLMARK_XENOBIOTIC_METABOLISM&fileType=grp)] | Genes encoding proteins involved in processing drugs & xenobiotics. | | 9 | 1.88 e^-7^ |
| [FATTY_ACID_METABOLISM](http://software.broadinstitute.org/gsea/msigdb/geneset_page.jsp?geneSetName=HALLMARK_FATTY_ACID_METABOLISM) [[158](http://software.broadinstitute.org/gsea/msigdb/download_geneset.jsp?geneSetName=HALLMARK_FATTY_ACID_METABOLISM&fileType=grp)] | Genes encoding proteins involved in metabolism of fatty acids. | | 8 | 3.52 e^-7^ |

**C. Suppressed by HFD in KO mice in the absence of Pio**: No overlaps found.

**D. Suppressed by HFD in the presence of Pio**: No overlaps found

*Using the Broad Institute platform with the Hallmark Gene Sets platform to identify the top 10 categories with p < 1e^-6^ (see text for details).

1. Landt, S. G., Marinov, G. K., Kundaje, A., Kheradpour, P., Pauli, F., Batzoglou, S., Bernstein, B. E., Bickel, P., Brown, J. B., Cayting, P., Chen, Y., DeSalvo, G., Epstein, C., Fisher-Aylor, K. I., Euskirchen, G., Gerstein, M., Gertz, J., Hartemink, A. J., Hoffman, M. M., Iyer, V. R., Jung, Y. L., Karmakar, S., Kellis, M., Kharchenko, P. V., Li, Q., Liu, T., Liu, X. S., Ma, L., Milosavljevic, A., Myers, R. M., Park, P. J., Pazin, M. J., Perry, M. D., Raha, D., Reddy, T. E., Rozowsky, J., Shoresh, N., Sidow, A., Slattery, M., Stamatoyannopoulos, J. A., Tolstorukov, M. Y., White, K. P., Xi, S., Farnham, P. J., Lieb, J. D., Wold, B. J., and Snyder, M. (2012) ChIP-seq guidelines and practices of the ENCODE and modENCODE consortia. *Genome Res* **22**, 1813-1831

2. Marinov, G. K., Kundaje, A., Park, P. J., and Wold, B. J. (2014) Large-scale quality analysis of published ChIP-seq data. *G3 (Bethesda)* **4**, 209-223
